# Supplementary material for: Hospital Readmission Risk and Risk Factors of People with a Primary or Secondary Discharge Diagnosis of Diabetes
Source: J Clin Med. 2023 Feb 6;12(4):1274. doi: 10.3390/jcm12041274 (PMC9961750; doi:10.3390/jcm12041274)
Supplement: Supplementary file 1 [file jcm-12-01274-s001.zip › jcm-2102285-supplementary.pdf]

Table S1. Most common secondary ICD-9-CM codes among 4,027 patients with a primary discharge diagnosis of diabetes.

ICD-9-CM Code*	Description	n	%
401, 403	Essential hypertension, hypertensive kidney disease	423	5.8
038, 041, 486, 599, 682, 730	Infection (cellulitis, abscess, pneumonia, urinay tract infection, septicemia, bacterial, osteomyelitis)	389	5.3
584, 585	Acute kidney failure, chronic kidney disease	378	5.1
V58	Radiotherapy, chemotherapy, transfusion, procedure aftercare, long-term medication use	309	4.2
305	Abuse of drugs or alcohol	273	3.7
276	Disorders of fluid electrolyte and acid-base balance	261	3.6
272	Disorders of lipid metabolism	246	3.3
707	Chronic ulcer of skin	235	3.2
410, 414, 440, 786	Ischemic heart disease, atherosclerosis, chest pain, or shortness of breath	208	2.8
V15	History presenting hazards to health (allergy, surgery, irradiation, Psychological trauma, injury, noncompliance, tobacco use, fall	195	2.7
357	Inflammatory and toxic neuropathy, Including polyneuropathy and diabetes	184	2.5
V45, 996, 998	Procedure or postoperative complication or postprocedure state	162	2.2
530	Diseases of esophagus, including reflux	125	1.7
285	Anemias	124	1.7
428	Heart failure	104	1.4
491, 493, 518	Lung diseases including asthma and chronic bronchitis	83	1.1

ICD-9-CM = International Classification of Diseases, Ninth Revision, Clinical Modification.

*All digits of ICD codes were included in the counts. The first 3 digits are presented here to consolidate the information.

Table S2. Most common reasons for hospital admission based on primary ICD-9-CM code among 4,027 patients with a secondary discharge diagnosis of diabetes.

ICD-9-CM Code*	Description	n	%
410, 414, 440, 786	Ischemic heart disease, atherosclerosis, chest pain, or shortness of breath	543	13.5
038, 041, 486, 599, 682, 730	Infection (cellulitis, abscess, pneumonia, urinay tract infection, septicemia, bacterial, osteomyelitis)	339	8.4
428	Heart failure	285	7.1
493, 491, 518	Lung diseases including asthma and chronic bronchitis	211	5.2
996, 998	Procedure or postoperative complication	157	3.9
433, 434	Ischemic stroke	107	2.7
584	Acute kidney failure	98	2.4
780	Alteration of consciousness, hallucinations, syncope, convulsions, dizziness, fever, or malaise	94	2.3
427	Cardiac dysrhythmias	91	2.3
276	Disorders of fluid electrolyte and acid-base balance	76	1.9
278	Overweight, obesity and other hyperalimentation	76	1.9
401, 403	Essential hypertension, hypertensive kidney disease	72	1.8
577	Diseases of pancreas, including pancreatitis	47	1.2

ICD-9-CM = International Classification of Diseases, Ninth Revision, Clinical Modification.
*All digits of ICD codes were included in the counts. The first 3 digits are presented here to consolidate the information.

Table S3. Risk factors for readmission in subgroup of patients with a primary discharge diagnosis of diabetes and an HbA1c value (n=2,182), OR (95%CI)

Predictor	Odds Ratio (95% CI)	P value
HbA1c, %	0.98 (0.93-1.03)	0.48
Outpatient visit (vs. Yes)		
No	5.94 (4.34-8.13)	<.0001
Not documented	0.33 (0.22-0.50)	<.0001
Diabetes inpatient consultation	0.69 (0.52-0.91)	0.009
Charlson comorbidity index (vs. 0)		
>6	1.35 (0.78-2.35)	0.28
5 – 6	1.05 (0.63-1.74)	0.85
3 – 4	1.30 (0.77-2.19)	0.32
1 – 2	1.78 (1.21-2.60)	0.003
Length-of-stay (days)(log)	1.14 (0.93-1.39)	0.21
Employment status (vs. Employed)		
Disabled	1.94 (1.14-3.31)	0.014
Retired	1.35 (0.77-2.37)	0.29
Unemployed	1.61 (0.95-2.73)	0.075
Other or unknown	0.96 (0.32-2.86)	0.94
Insurance status (vs. Private)		
Medicaid	0.88 (0.57-1.34)	0.54
Medicare	0.85 (0.58-1.26)	0.43
None	0.28 (0.12-0.64)	0.003
Educational level (vs. College graduate)		
Less than high school	1.27 (0.75-2.16)	0.38
Any high school	1.13 (0.75-1.72)	0.56
Some college	1.92 (1.08-3.41)	0.026
Not recorded	0.97 (0.51-1.85)	0.93
Discharged within 90 days before admission	1.69 (1.28-2.25)	0.0003
Pre-admission thiazolidinedione use	0.56 (0.28-1.11)	0.096
Gastroparesis ever	1.79 (1.22-2.63)	0.003
Anemia ever	1.29 (0.97-1.72)	0.078

Table S4. Risk factors for readmission in subgroup of patients with a secondary discharge diagnosis of diabetes and an HbA1c value (n=1,492), OR (95%CI)

Predictor	Odds Ratio (95% CI)	P value
HbA1c, %	0.97 (0.89-1.06)	0.53
Outpatient visit (vs. Yes)		
No	6.16 (3.99-9.53)	<.0001
Not documented	0.55 (0.34-0.90)	0.018
Discharge status of index admission (vs. Home)		
Against medical advice	3.99 (1.74-9.14)	0.001
Home with nursing care	1.29 (0.84-1.98)	0.25
Sub-acute facility	1.37 (0.87-2.16)	0.17
Length-of-stay (days)(log)	1.11 (0.89-1.39)	0.34
Employment status (vs. Employed)		
Disabled	3.14 (1.37-7.23)	0.007
Retired	2.64 (1.16-5.98)	0.020
Unemployed	2.37 (1.03-5.43)	0.041
Other or unknown	2.21 (0.69-7.06)	0.18
Insurance status (vs. Private)		
Medicaid	1.31 (0.70-2.47)	0.40
Medicare	1.04 (0.60-1.81)	0.89
Discharged within 90 days before admission	2.10 (1.48-2.98)	<.0001
Pancreatitis ever	2.43 (1.26-4.68)	0.008
Serum sodium (vs. Normal)		
Low <135 mmol/L	1.37 (0.84-2.25)	0.21
High >145 mmol/L	2.50 (0.50-12.61)	0.27
Urgent or emergent admission	2.51 (1.18-5.35)	0.017
Serum albumin (vs. ≥40 g/L)		
<40 g/L	1.66 (1.12-2.46)	0.012
Unknown	1.13 (0.61-2.12)	0.69
Anemia ever	1.60 (1.12-2.31)	0.011
